# Prediction of methylation status using WGS data of plasma cfDNA for multi-cancer early detection (MCED)

**DOI:** 10.1186/s13148-024-01646-6

**Published:** 2024-02-27

**Authors:** Pin Cui, Xiaozhou Zhou, Shu Xu, Weihuang He, Guozeng Huang, Yong Xiong, Chuxin Zhang, Tingmin Chang, Mingji Feng, Hanming Lai, Yi Pan

**Affiliations:** 1 Shenzhen Rapha Biotechnology Incorporate, Shenzhen, 518118 China; 2 Department of Liver Disease, Shenzhen Traditional Chinese Medicine Hospital, Shenzhen, 518033 China; 3 Department of Oncology, Shenzhen Hospital, University of Chinese Academy of Sciences, Shenzhen, 518106 China; 4 Department of Hepatobiliary Gastrointestinal Surgery, Shenzhen Hospital, University of Chinese Academy of Sciences, Shenzhen, 518106 China; 5 Department of Gastroenterology, Shenzhen Hospital, University of Chinese Academy of Sciences, Shenzhen, 518106 China; 6 Department of Endoscopy, The First Affiliated Hospital of Xinxiang Medical University, Weihui, 453100 China; 7 Shenzhen Institute of Advanced Technology, Chinese Academy of Sciences, Shenzhen, 518055 China

**Keywords:** Methylation status, Multi-cancer early detection, WGS, WGBS, cfDNA fragmentation profile

## Abstract

**Background:**

Cell-free DNA (cfDNA) contains a large amount of molecular information that can be used for multi-cancer early detection (MCED), including changes in epigenetic status of cfDNA, such as cfDNA fragmentation profile. The fragmentation of cfDNA is non-random and may be related to cfDNA methylation. This study provides clinical evidence for the feasibility of inferring cfDNA methylation levels based on cfDNA fragmentation patterns. We performed whole-genome bisulfite sequencing and whole-genome sequencing (WGS) on both healthy individuals and cancer patients. Using the information of whole-genome methylation levels, we investigated cytosine–phosphate–guanine (CpG) cleavage profile and validated the method of predicting the methylation level of individual CpG sites using WGS data.

**Results:**

We conducted CpG cleavage profile biomarker analysis on data from both healthy individuals and cancer patients. We obtained unique or shared potential biomarkers for each group and built models accordingly. The modeling results proved the feasibility to predict the methylation status of single CpG sites in cfDNA using cleavage profile model from WGS data.

**Conclusion:**

By combining cfDNA cleavage profile of CpG sites with machine learning algorithms, we have identified specific CpG cleavage profile as biomarkers to predict the methylation status of individual CpG sites. Therefore, methylation profile, a widely used epigenetic biomarker, can be obtained from a single WGS assay for MCED.

**Supplementary Information:**

The online version contains supplementary material available at 10.1186/s13148-024-01646-6.

## Introduction

Cell-free DNA fragmentomics is a rapidly developing emerging field with significant biological and clinical implications. Recently, it has been mainly used as an epigenetic marker for early detection of cancers [1, 2]. The fragmentation of cfDNA is related to the nucleosome packaging pattern of chromosomal DNA [3, 4], hence is related to gene expression profile. For example, compared to that of healthy individuals, cfDNA fragmentation profile of cancer patients shows a relatively lower peak at 166 bp but a higher proportion of short fragments below 150 bp in cfDNA fragment size. Additionally, the fragmentation patterns of cfDNA molecules contain a wealth of molecular information related to their tissue of origin [5].

Conventionally, the detection of DNA methylation status requires bisulfite conversion in the initial step to chemically convert the unmethylated cytosines to uracils and to be further detected as an thymine in subsequent amplification and sequencing using a non-proofreading polymerase, while the methylated cytosines remain intact [6] However, a major drawback of this method is the bisulfite conversion [7], a harsh chemical reaction which requires intensive laboratory work, causes severe damage to DNA molecules and thus affects the detection of low-abundance target molecules(such as tumor cfDNA in early-stage cancer patients). Although technology developments like enzymatic conversion [8] and direct methyl-sequencing on third- generation sequencing platforms [9, 10] have reduced or even avoided this impact, bisulfite conversion-based techniques are still the most widely used method for methylation detection and WGBS is generally regarded as the gold standard for methylation sequencing, especially in clinical applications [11, 12].

Previous studies have revealed that the cleavage profile of cfDNA was non-random and was correlated with the methylation status [13–15]. If this hypothesis can be confirmed and be further validated in WGS data, this method can simultaneously avoid the use of bisulfite conversion and the high cost of bisulfite sequencing with subsequent complicated methylation data analysis [16], which can easily be integrated into WGS assay, a labor- and cost-efficient routine genomic test [17, 18].

In this study, we collected data of WGS and WGBS from healthy individuals and cancer patients to investigate the methylation status of CpG sites surrounding cfDNA cleavage profile (within an 11-nucleotide [nt] window). By analyzing the cleavage profile of hyper- and hypo-methylated CpG sites in WGBS data, we trained an optimized model using machine learning algorithms to predict the methylation status of an individual CpG site. And based on this model, we extracted CpG cleavage profile from WGS data and trained an optimal model to predict the methylation status of CpG sites.

## Methods

### Participant recruitment and study design

**Fig. 1:**
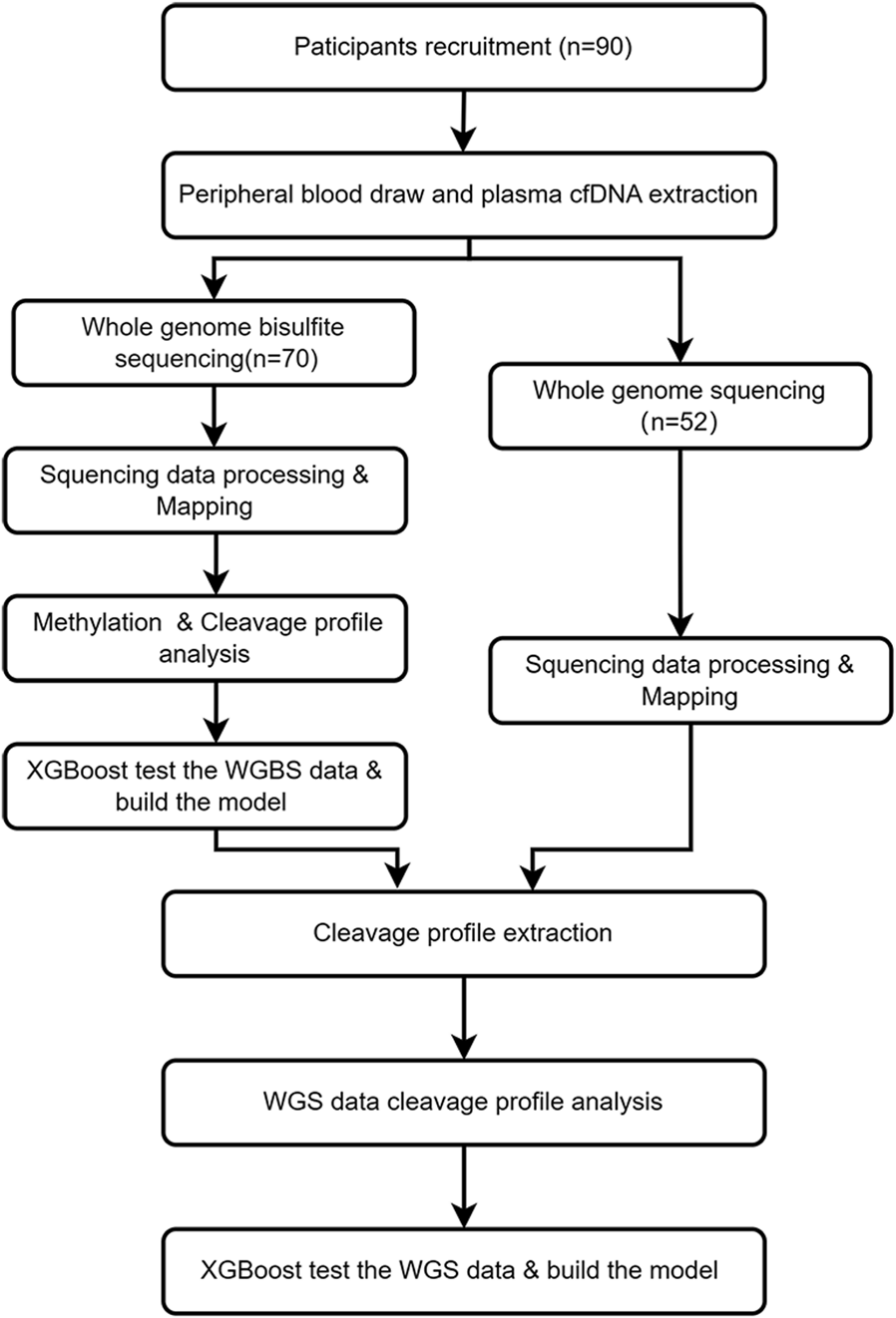
The procedure for the methylation analysis of cfDNA fragment distribution

Totally, 90 participants were recruited into this study, including 17 healthy individuals, 26 hepatocellular carcinoma (HCC) patients, 32 lung cancer patients, and 15 colorectal cancer patients (Additional file 5: Table S1). And among them, 32 participants, including 7 healthy individuals, 12 HCC patients, 2 lung cancer patients, and 11 colorectal cancer patients, were proceeded with both WGBS and WGS. Therefore, there were 70 samples in WGBS dataset and 52 samples in WGS dataset (Fig. 1). Protocol for healthy individuals and cancer patient sample collection of this study was approved by the ethics committee of Shenzhen Traditional Chinese Medicine Hospital with Approval No. 201858 and protocols for all other types of cancer sample collection were approved by the ethics committee of University of Chinese Academy of Sciences Shenzhen Hospital with Approval No. LL-KT-2022053. All of them were performed following international standards of good clinical practice. All HCC patients were enrolled at the time of diagnosis in the Shenzhen Traditional Chinese Medicine Hospital from March to December 2021, while patients with other cancer types were enrolled at the time of diagnosis in the University of Chinese Academy of Sciences Shenzhen Hospital from May to December 2022.

### Cell-free DNA extraction from plasma samples

Plasma was obtained by centrifugation of whole blood at 1600*g* for 10 min. And then the supernatant was transferred to a new tube and further centrifuged at 10,000*g* for 15 min to remove cell debris from the plasma. For each participant, cfDNA was isolated and purified from 3 mL plasma using the HiPure Circulating DNA Midi Spin Kit S (Magen Biotech Inc., Guangzhou, China) into a final elution volume of 50 μL. Quality control was performed to these libraries using Qsep100 (Bio-optic. Inc., Taiwan, China) for fragment size distribution and Qubit 4.0 (ThermoFisher Inc., MA, USA) for concentration, and cfDNA samples with abnormal fragment size distribution (showing distribution outside the normal cfDNA peak) and ultra-high concentration were identified as contaminated with genomic DNA (mainly from deceased white blood cells during logistics).

### WGBS library construction and sequencing

For the cohort of participants including 12 healthy individuals, 12 HCC patients, 26 lung cancer patients, and 20 colorectal cancer patients, WGBS was performed with 10–20 ng of cfDNA input per participant. WGBS Library for whole-genome bisulfite sequencing was constructed for each cfDNA sample using RainbowMerry cfDNA Methylseq Library Prep Kit (Rapha Biotech. Inc., Shenzhen, China). This kit combined bisulfite conversion and single-stranded NGS library preparation technology [19] with modifications including that usage of SA-coated beads was omitted and Klenow fragment was replaced by a uracil tolerant enzyme with 5′–3′ synthesis activity. This WGBS library construction method was more efficient than conventional WGBS library construction methods in terms of converting more original DNA molecules into sequencing library after bisulfite conversion. It allowed library preparation and sequencing with ultra-low DNA input suitable for cfDNA. Quality control was performed to these libraries using Qsep100 (Bio-optic. Inc., Taiwan, China) and Qubit 4.0 (ThermoFisher. Inc., MA, USA), and every four libraries were sent for sequencing on a separate lane of MGI-2000 sequencer using DNBSEQ™ technology to guarantee that the average sequencing depth of each sample reaches at least 8.74 × with 12.24 × on average.

### Whole-genome sequencing library construction and sequencing

For the cohort of participants including 12 healthy individuals, 12 HCC patients, 14 lung cancer patients, and 14 colorectal cancer patients, WGS was performed using 10 ng of cfDNA input for each participant. WGS libraries were constructed using RainbowOne Universal DNA Library Prep Kit for MGI (Rapha Biotechnology Inc., China), following the fundamental principles for WGS library preparation including molecular end repair, sequencing adaptor ligation, and library clean up. The libraries were then amplified using VAHTS HiFi Amplification Mix (cat. N616-01) and purified using VAHTS DNA Clean Beads (cat. N411-02), both purchased from Vazyme Biotech Co., Ltd., Nanjing, China. Quality control was performed on these libraries using Qsep100 (Bio-optic. Inc., Taiwan, China) and Qubit 4.0 (ThermoFisher. Inc., MA, USA), and libraries were sent for sequencing on MGI-2000 sequencer (BGI Genomics Inc., Wuhan, China) using DNBSEQ™ technology and sequencing mode. The sequencing quote was designed to ensure that the average sequencing depth of each sample reaches at least 14.72 × with 18.34 × on average.

### Statistical analysis and machine learning

We used XGBoost [20] as the machine learning algorithm to build our models and performed the feature extraction to WGBS data as follows. Firstly, adapter sequences, low-quality bases, and short-length sequences were trimmed off from the raw WGBS data using the software fastp. Then, the filtered reads were aligned to the reference genome (GRCh38) using the software bsbolt, followed by sorting and PCR duplicate removal of the aligned BAM file. Next, the CpG site information and methylation expression information were extracted using the software MethyDackel, with a 5nt length range upstream and downstream of the methylated C position as the CpG cleavage windows. The relative positions of this sequence corresponded to − 5, − 4, − 3, − 2, − 1, 0, + 1, + 2, + 3, + 4, and + 5. The number of broken reads at each base position within the CpG cleavage windows was calculated. The CpG cleavage windows, with a depth greater than 50 × and a terminal sequence count greater than 10 at the methylated C position (position 0), were selected for subsequent model training. The hyper- and hypo-methylated CpG sites were defined as CpG sites with a methylation index > 70% and < 30%, respectively. A 11-nt length CpG cleavage window was used as the unit, and the cleavage proportion was calculated for each nt within the window to form the CpG cleavage profile, as shown in the following formula:

$${\text{Cleavage}}\,{\text{proportion}}\,{\text{at}}\,{\text{site}}\,i = {\text{No}}.\,{\text{of}}\,{\text{fragment}}\,{\text{ends}}\,{\text{at}}\,{\text{a}}\,{\text{ste}}\,i/{\text{sequening}}\,{\text{depth}}\,{\text{at}}\,{\text{site}}\,i$$

The hypo-methylated CpG cleavage profiles were labeled as CLASS0, while the hypermethylated ones were labeled as CLASS1. The CpG cleavage profile model consists of 11 features, which were the cleavage proportions of each of the 11-nt cleavage window. Each CpG cleavage profile was used as data input for building models. The dataset was randomly divided into a training set and a validation set at a ratio of 7:3, and the model was calculated multiple times until a steady result was achieved.

For WGS dataset, we firstly performed quality control of the raw data using the software fastp. Then, we aligned the filtered reads to the reference genome (GRCh38) using the software bwa, followed by sorting and PCR duplicate removal of the aligned BAM file. Based on the hyper- and hypo-methylated information of CpG cleavage windows from the WGBS data of the same group of samples, we extracted, calculated, and classified the cleavage proportion of CpG cleavage windows from the WGS data's BAM files. And based on the cleavage profile from WGS data, we trained and validated the model to predict the methylation status of an individual CpG site in cfDNA.

The modeling for each group of WGBS and WGS data was iteratively trained to obtain the optimal result. The CpG cleavage windows from the training set of the optimal model were selected as candidate biomarkers for functional enrichment analysis.

Based on true-positive (TP), true-negative (TN), false-positive (FP), and false-negative (FN) of cancer prediction, the sensitivity [TP/(TP + FN)], specificity [TN/(TN + FP)], true-positive rates (TPR) [TP/(TP + FN)], and false-positive rate (FPR) [FP/(FP + TN)] predictive values, accuracy [(TP + TN)/(TP + FP + TN + FN)], were calculated using the Numpy (v 1.18.5).

## Results

### Verification of the direct impact of DNA methylation on the cleavage profile of cfDNA

To verify if the level of DNA methylation affects the cleavage profile of cfDNA, we performed WGBS to 70 samples, including 12 healthy individuals, 12 HCC patients, 26 lung cancer patients, and 20 colorectal cancer patients. And we also performed WGS to 52 samples, including 12 healthy individuals, 12 HCC patients, 14 lung cancer patients, and 14 colorectal cancer patients (Fig. 1). To obtain a sufficient depth of sequencing for the analysis of cleavage proportion, we merged the samples of the same group in WGBS dataset, and named them A12, H12, L26, and C20, respectively (Additional file 6: Table S2). The number of cleavage windows for hyper- and hypo-methylated CpG sites in A12 was 22,271,691 and 579,870, respectively. The number of cleavage windows for hyper- and hypo-methylated CpG sites in H12 was 20,299,374 and 704,054, respectively. The number of cleavage windows for hyper- and hypo-methylated CpG sites in L26 was 22,613,911 and 614,383, respectively. The number of cleavage windows for hyper- and hypo-methylated CpG sites in C20 was 22,319,554 and 574,260, respectively. The mean cleavage proportion for hyper- and hypo-methylated CpG sites in A12 were 0.0329 and 0.0276, respectively, with a difference of approximately 1.2 times. The mean cleavage proportion for hyper- and hypo-methylated CpG sites in H12 were 0.1354 and 0.0841, respectively, with a difference of approximately 1.6 times. The mean cleavage proportion for hyper- and hypo-methylated CpG sites in L26 were 0.2368 and 0.1152, respectively, with a difference of approximately 2.0 times. The mean cleavage proportion for hyper- and hypo-methylated CpG sites in C20 were 0.1924 and 0.0914, respectively, with a difference of approximately 2.1 times. Comparing to that of healthy individuals, the difference of cleavage proportions between hyper- and hypo-methylated CpG sites in three groups of cancer datasets was more pronounced. The analysis process is illustrated in Fig. 1.

**Fig. 2:**
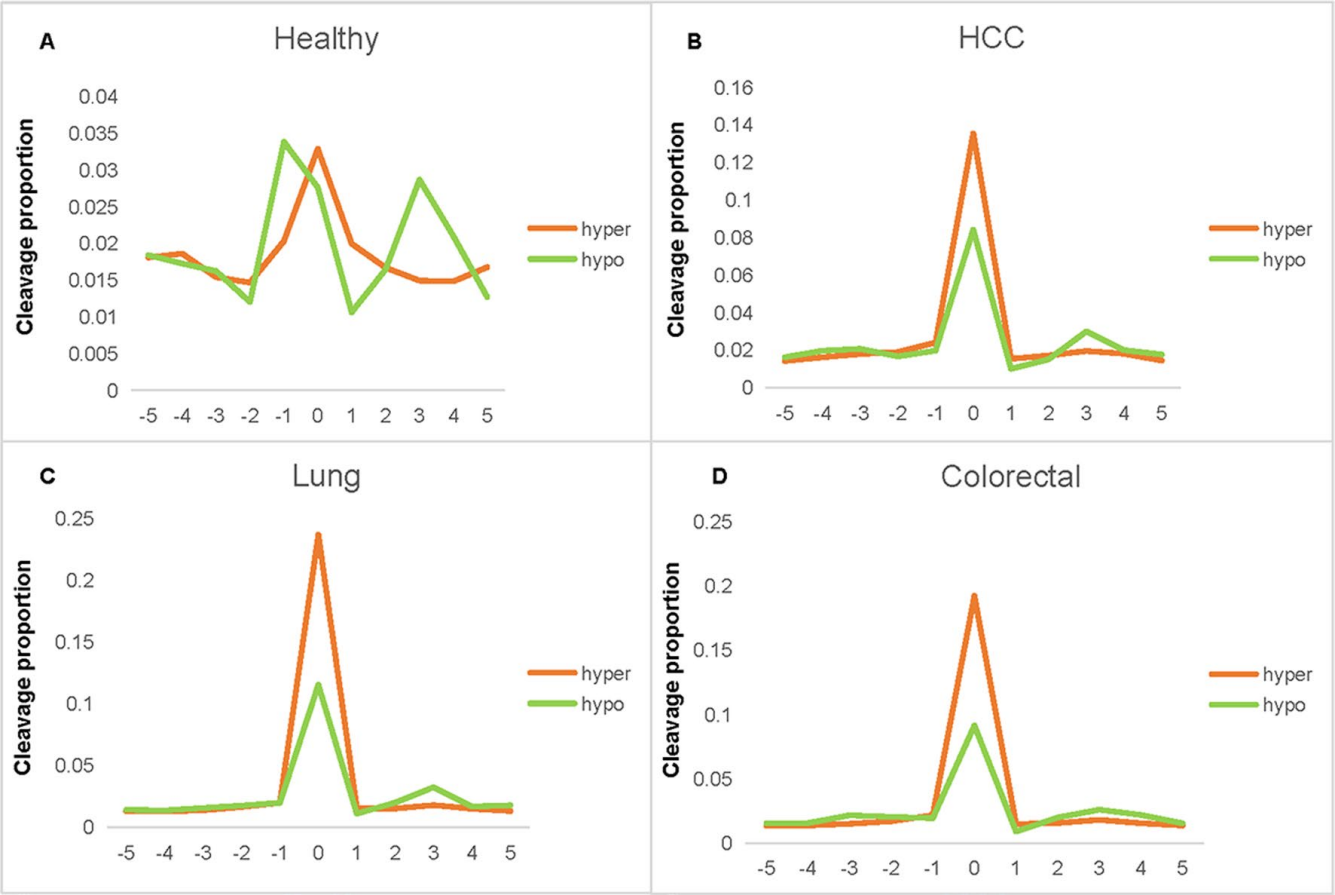
Cleavage profile of cfDNA at CpG sites. **a** The cleavage profile of cfDNA for healthy individuals; **b** the cleavage profile of cfDNA for HCC patients, C: the cleavage profile of cfDNA for lung cancer patients, **d** the cleavage profile of cfDNA for colorectal cancer patients

In healthy individuals and cancer patients, the mean cleavage proportion of cytosine in hyper- methylated CpG sites is 0.2–1.1 times higher than that of hypo-methylated CpG sites. And the mean cleavage proportion drops dramatically at the 1-nt position immediately preceding a methylated CpG as shown in Fig. 2. This difference in cleavage resulted in characteristic changes in the relative presentation of CGN and NCG motifs at the sequence terminus, where N represents any nucleotide. The ratio of CGN/NCG motifs was related to the methylation level of CpG sites. Therefore, cfDNA cleavage profile can be used to analyze cfDNA methylation status.

### Prediction models for methylation status at CpG sites using cleavage profile from WGBS data

We selected CpG site cleavage windows with sequencing depth greater than 50 × and a terminal sequence count greater than 10 for model training and validation, to ensure there are effective cleavage proportion to be used by the model’s machine learning algorithm. Based on the CpG cleavage proportion, prediction model was trained and validated using the machine learning algorithm, XGBoost, to identify a CpG site as being hyper- or hypo-methylated.

**Fig. 3:**
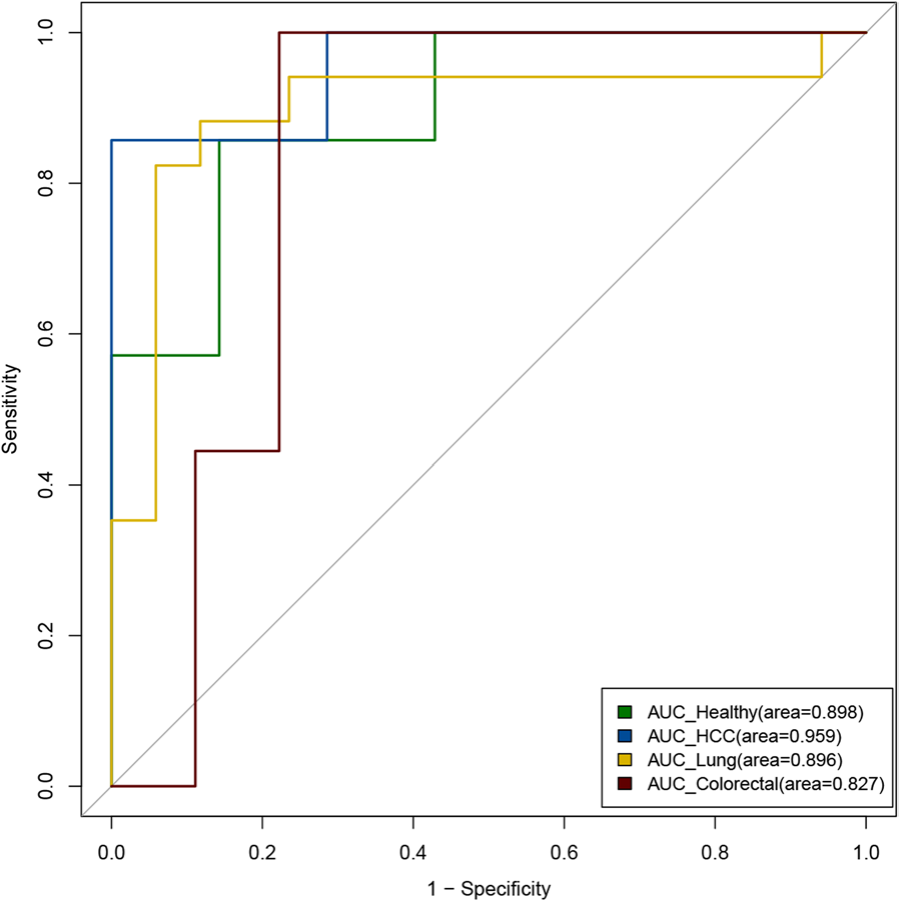
The AUC curves of the prediction models for methylation status using WGBS data. Curves in different colors represent different groups of participants, with green for healthy individuals, blue for HCC patients, yellow for lung cancer patients, and purple for colorectal cancer patients

The AUC of the prediction model for healthy individuals was 0.898, with a sensitivity of 0.857 and a specificity of 0.857. The AUC of the prediction model for patients with HCC was 0.959, with a sensitivity of 0.857 and a specificity of 1. The AUC of the prediction model for patients with lung cancer was 0.896, with a sensitivity of 0.882 and a specificity of 0.823. The AUC of the prediction model for patients with colorectal cancer was 0.827, with a sensitivity of 1 and a specificity of 0.777. The modeling results proved the feasibility to predict the methylation status of individual CpG sites using cfDNA cleavage profile. And the performance of four models shows they are not affected by the pathological conditions of the participants (Fig. 3).

### Prediction models for methylation status at CpG sites using cleavage profile from WGS data

Similar to the process of setting up prediction models using the WGBS data, we extracted the optimal cleavage windows from WGBS data and calculated cleavage proportion for each of these windows using WGS data. These values of cleavage proportion were then used as input to build models via XGBoost algorithm to predict the methylation status of CpG sites in these windows. To obtain a sufficient depth of sequencing for the analysis of cleavage proportion, we merged the samples of the same group in WGS dataset, and named them A12, H12, L14, and C14, respectively (Additional file 6: Table S2).

**Fig. 4:**
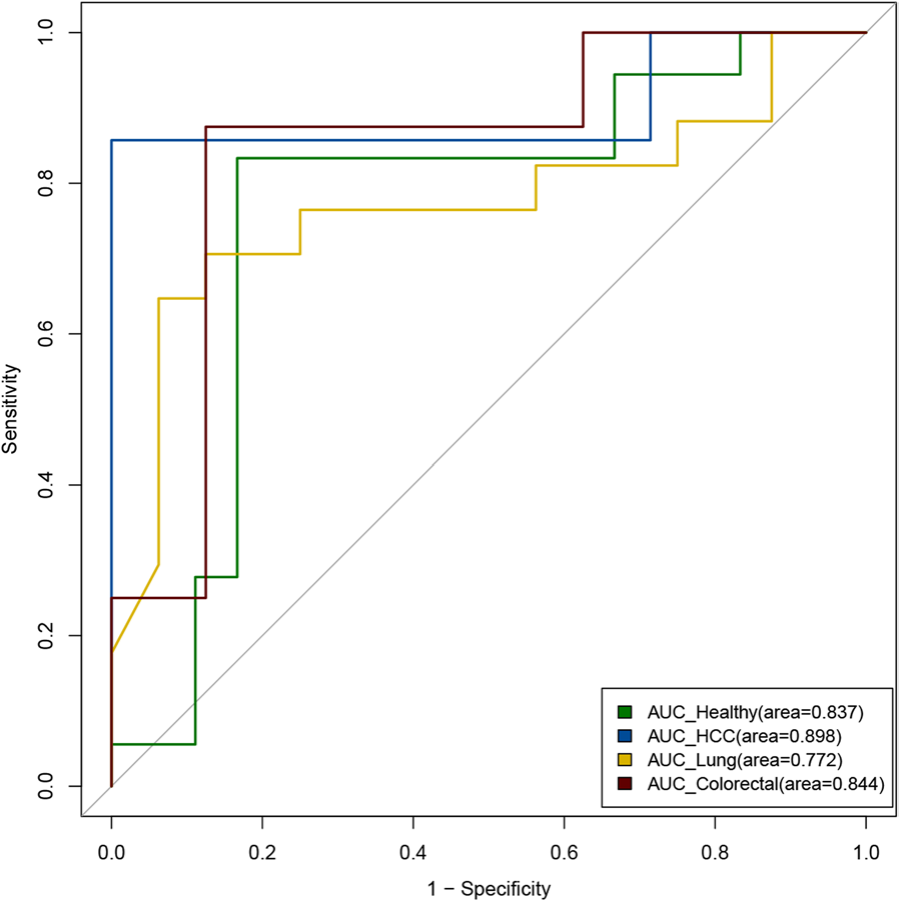
The AUC curves of the prediction models for methylation status using WGS data. Curves in different colors represent different groups of participants, with green for healthy individuals, blue for HCC patients, yellow for lung cancer patients, and purple for colorectal cancer patients

The AUC of the prediction model for healthy individuals was 0.837, with a sensitivity of 1 and a specificity of 0.714. The AUC of the prediction model for patients with HCC was 0.898, with a sensitivity of 0.857 and a specificity of 0.857. The AUC of the prediction model for patients with lung cancer was 0.772, with a sensitivity of 0.764 and a specificity of 0.750. The AUC of the prediction model for patients with colorectal cancer was 0.844, with a sensitivity of 0.750 and a specificity of 0.857. The modeling results proved the feasibility to predict the methylation status of individual CpG sites using cfDNA cleavage profile solely using WGS data. And the performance of four models shows they are not affected by the pathological conditions of the participants as shown in Fig. 4.

### Functional annotation of optimal cleavage windows for biomarker development

**Fig. 5:**
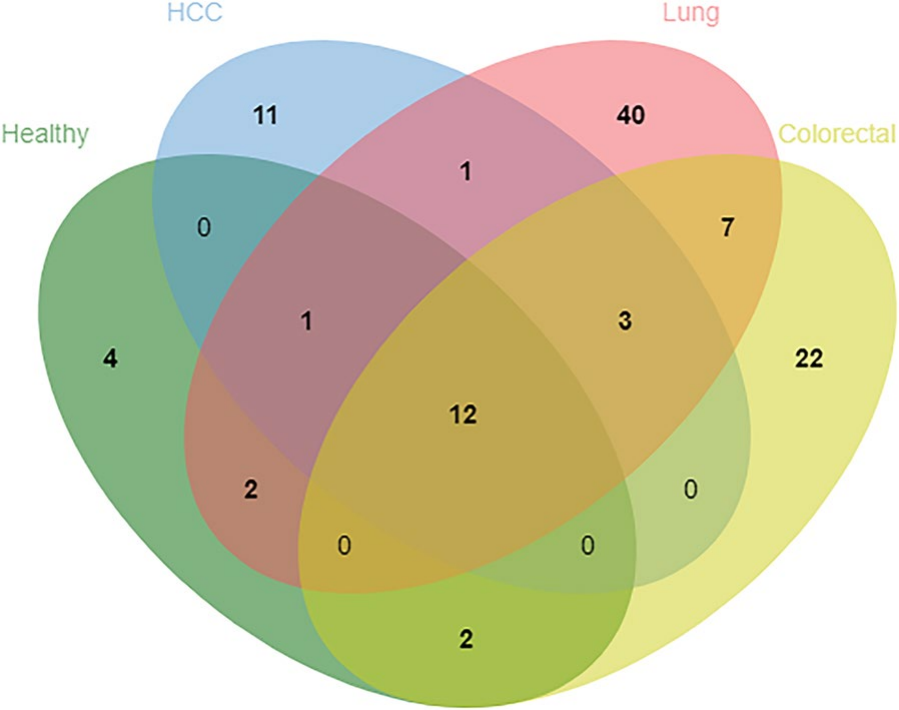
Numbers of shared and unique candidate biomarkers (genes) among four groups of participants. The oval in green represents the genes identified in the group of healthy individuals; the oval in light blue represents the genes identified in the group of HCC patients; the oval in pink represents the genes identified in the group of lung cancer patients; and the oval in yellow represents the genes identified in the group of colorectal cancer patients

From the prediction models above, we obtained the optimal cleavage windows of each groups of participants and located their positions in genome to identify related genes. Then, we performed function enrichment annotation [21] to these genes and compared their occurrence between different groups of participants. Consequently, part of these genes were selected into three categories of potential biomarkers (Fig. 5).

**Fig. 6:**
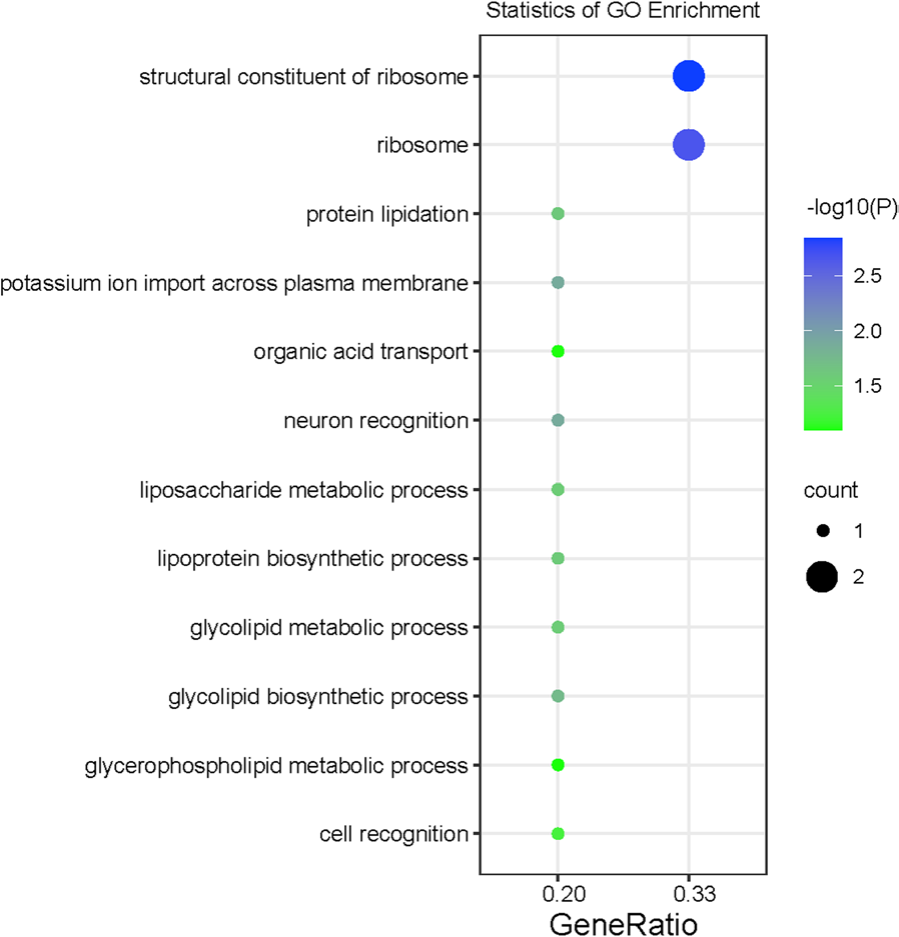
Functional annotation of 12 genes shared among all four groups

Firstly, there are 12 genes shared among all four groups of participants and functional annotation of these genes indicated that they were significantly involved in the pathways with fundamental biological functions like composition of ribosome, lipidation of proteins, and transmembrane transport (Fig. 6). And these genes could serve as potential biomarkers in a general model to predict methylation status based on cleavage profile from WGS data.

The horizontal axis represents the proportion of genes enriched to the target pathway in the total number of genes, and the vertical axis represents the pathway of GO enrichment analysis. The bluer the color of the dot is, the more significant the probability is for the genes to be annotated to this GO Term.

Secondly, there are three genes shared among the three groups of cancer patients but not with the group of healthy individuals, namely *BAGE2, HAVCR1P1,* and *LINC01667.* Numerous studies have reported the correlation of the abnormal expression of *BAGE2* [22, 23] and *LINC01667* [24] with carcinogenesis of multiple types of cancers. Therefore, these genes could be potential biomarkers to discriminate healthy individuals from cancer patients in general.

**Fig. 7:**
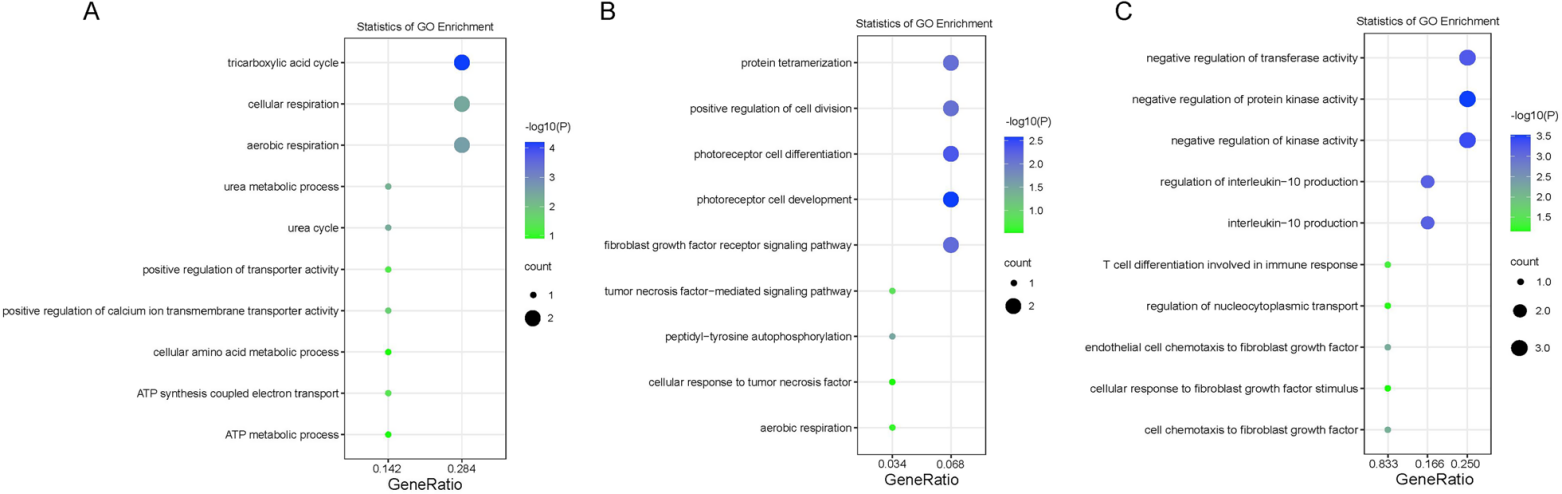
Functional annotation of genes unique to each of the three types of cancers. **a** For the group of patients with HCC; **b** For the group of patients with Lung cancer; **c** For the group of patients with Colorectal cancer

Thirdly, there are 11 genes unique to the group of HCC patients, 40 genes unique to the group of lung cancer patients, and 22 genes unique to the group of colorectal patients. And functional annotation of these genes showed their involvement in multiple pathways related with tumorigenesis (Fig. 7). Furthermore, some of the genes were identified to be associated with organ-specific functions, e.g., the GO terms urea metabolic process and urea cycle identified in the annotation result of genes unique to HCC patients’ group (Fig. 7a), which suggested they could be potential biomarkers specifically for detection of each of the related types of cancer.

The GO directed acyclic graphs related to GO terms in Figs. 6, 7a, b, c were illustrated in Additional file 1, 2, 3, 4: Figs. S1, S2, S3, and S4, respectively. And the GO Term information was listed in Additional file 7: Table S3.

## Discussion

In this study, we validated the correlation between the cleavage profile of CpG sites and the methylation status of C sites in cfDNA WGBS datasets of patients with three common types of solid tumors, including HCC, lung cancer, and colorectal cancer. And based on this assay, we established the method to predict methylation status of individual CpG sites in cfDNA using cleavage profile solely inferred from WGS data. Furthermore, we performed function annotation to the cleavage windows from the optimal prediction model above to identify related genes and their biological functions. And we compared the genes from the three types of cancer patients’ groups and healthy individuals’ group to identify shared and unique ones between these groups for biomarker development. This novel method of methylation analysis does not require labor and cost intensive experiment with bisulfite or enzyme-catalyzed cytosine conversion, which is a necessary step in most routine methods for methylation detection. Therefore, our approach not only preserves the integrity of the DNA molecules, but also allows simultaneous analysis of genetic and epigenetic information in a single WGS assay, opening new possibilities for large-scale methylation research and translation.

We used a machine learning algorithm to train a model on 11-nt cleavage windows centered on CpG. Different cleavage windows and the cleavage values at each position in the window form a numerical matrix. Through model training with the XGBoost algorithm, the optimal model can effectively distinguish between hyper- and hypo-methylated levels of CpG sites in clinical samples, achieving AUCs of 0.959, 0.896, and 0.827 for the groups of patients with HCC, lung cancer, and colorectal cancer, respectively. And such high level of accuracy is enough for this method to be used in clinical application.

What is more, our study identified three genes (*BAGE2, HAVCR1P1, and LINC01667*) as potential biomarkers to discriminate healthy individuals from multiple types of cancers in general. Additionally, we also identified a number of genes unique to a specific type of each of the three cancers (Fig. 5). Therefore, these genes can serve as potential biomarkers for cancer type identification, a necessary component of multiple cancer early detection (MCED) using plasma cfDNA, in which the cfDNA molecules with cancerous aberrations could possibly originate from anywhere in the body where blood circulation reaches and thus it is a necessity to source these cancer-featured cfDNA molecules for their tissue or organ origin to identify cancer types, and ultimately for efficient diagnosis and treatment.

As the CpG cleavage windows in each set of WGS data for methylation status prediction were obtained through analysis of WGBS data in this study, there is a concern that the cleavage profile might be altered in bisulfite-treated DNA, so the relevance of using optimal cleavage windows from WGBS data to predict methylation status in naturally fragmented DNA (in WGS data) is uncertain. Several studies have suggested that bisulfite conversion can shorten large-size genomic DNA(generally over 20 kb in size) but does not affect small-sized cfDNA(mainly distributing in 140–170 bp size range) [25–28], neither does it affect the features in cfDNA end motifs [29]. Therefore, the influence of bisulfite conversion to the cleavage profile of cfDNA as presented in WGBS data is limited and should not affect the methodology of this study. However, the detailed chemical mechanism of bisulfite conversion’s influence to cleavage profile remains unclear and further study on this issue is needed, which is a limitation of this study.

Another limitation of this study is the sequencing cost. The CpG cleavage windows for model training currently need a C-site with a sequencing depth greater than 50× and a terminal sequence count greater than 10, which requires the average sequencing depth of WGS to be at least 50×. This results in high cost in sequencing and reduces the generalizability of the method in routine clinical application. However, this problem can be solved when sequencing cost continues to drop and the prediction models are further optimized to tolerate WGS data as input with depth lower than 50×.

In summary, we have established a cfDNA cleavage profile based model through machine learning algorithms, to predict methylation status of individual CpG sites in a minimum-invasive, labor and cost-efficient manner. And the potential biomarkers found in this study provides an idea for targeted sequencing of specific genomic regions relevant to selected cleavage windows, rather than sequencing on whole-genome scale, e.g., setting up a multi-cancer detection panel, which could reduce the cost and promote the applicability of our method. This study opens up avenues for technology and biomarker development for multi-cancer early detection, an emerging field with great impact on public health, and for other medical applications such as noninvasive prenatal screening and organ transplantation.

### Supplementary Information

**Additional file 1**. Figure S1. The GO directed acyclic graphs for the unique biomarker genes of HCC group.

**Additional file 2**. Figure S2. The GO directed acyclic graphs for the unique biomarker genes of Lung cancer group.

**Additional file 3**. Figure S3. The GO directed acyclic graphs for the unique biomarker genes of Colorectal cancer group.

**Additional file 4**. Figure S4. The GO directed acyclic graphs for the shared biomarker genes among three cancer groups.

**Additional file 5**. Table S1. Arrangement of WGBS and WGS for different types of participants.

**Additional file 6**. Table S2. QC statistics of data concatenation of four groups of participants.

**Additional file 7**. Table S3. GO Term interpretation of shared and unique biomarkers among three cancer groups.

## Data Availability

The sequencing data in this study are available from the corresponding authors upon request.

## Abbreviations

WGS: Whole-genome sequencing
WGBS: Whole-genome bisulfite sequencing
AUC: Area under the curve
cfDNA: Cell-free DNA
MCED: Multi-cancer early detection
