# Supplementary figures and images for: Prediction of methylation status using WGS data of plasma cfDNA for multi-cancer early detection (MCED)
Source: Clin Epigenetics. 2024 Feb 27;16:34. doi: 10.1186/s13148-024-01646-6 (PMC10898085; doi:10.1186/s13148-024-01646-6)

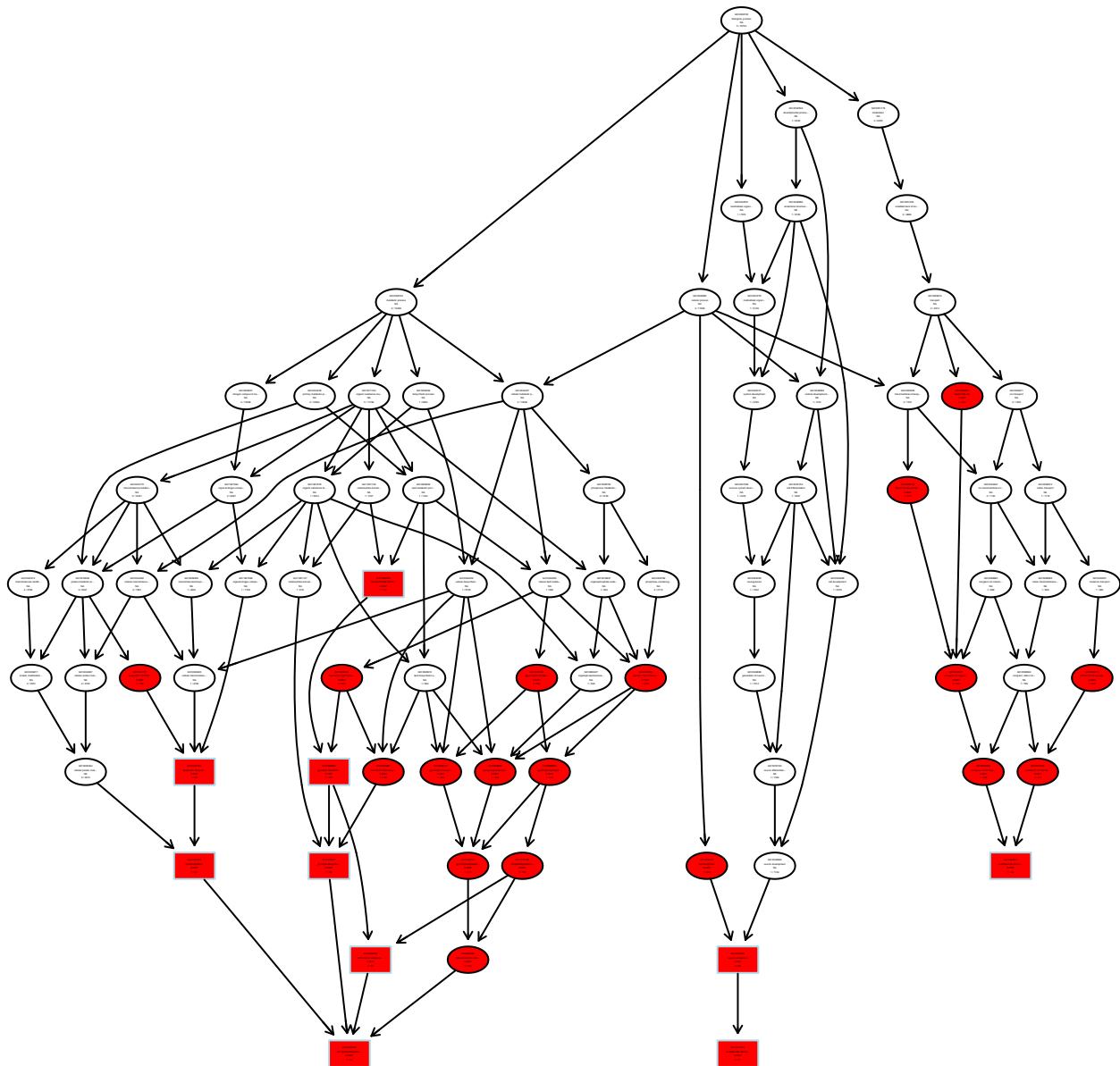

Supplement: Supplementary file 1 — Additional file 1. Figure S1. The GO directed acyclic graphs for the unique biomarker genes of HCC group. [file 13148_2024_1646_MOESM1_ESM.pdf]

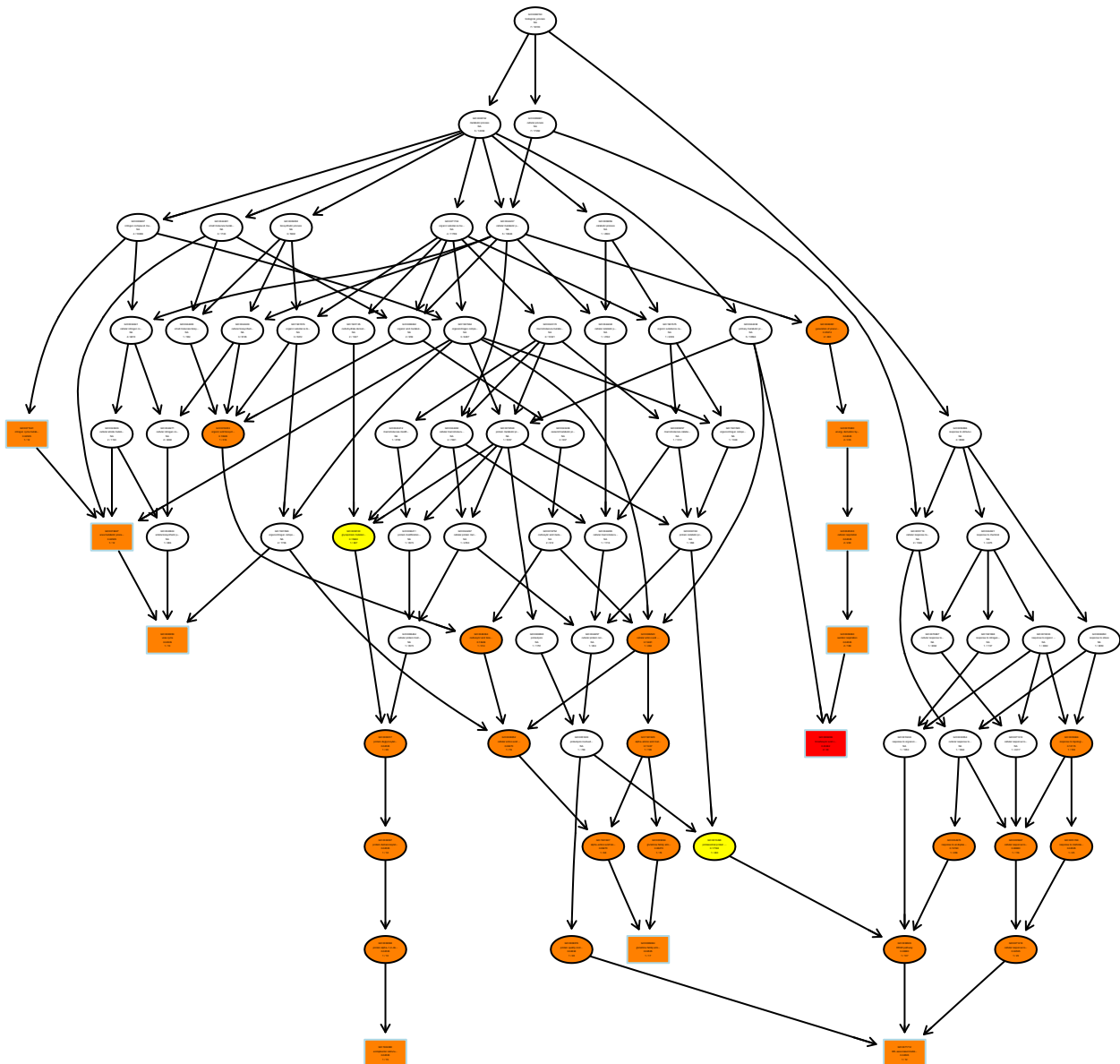

Supplement: Supplementary file 2 — Additional file 2. Figure S2. The GO directed acyclic graphs for the unique biomarker genes of Lung cancer group. [file 13148_2024_1646_MOESM2_ESM.pdf]

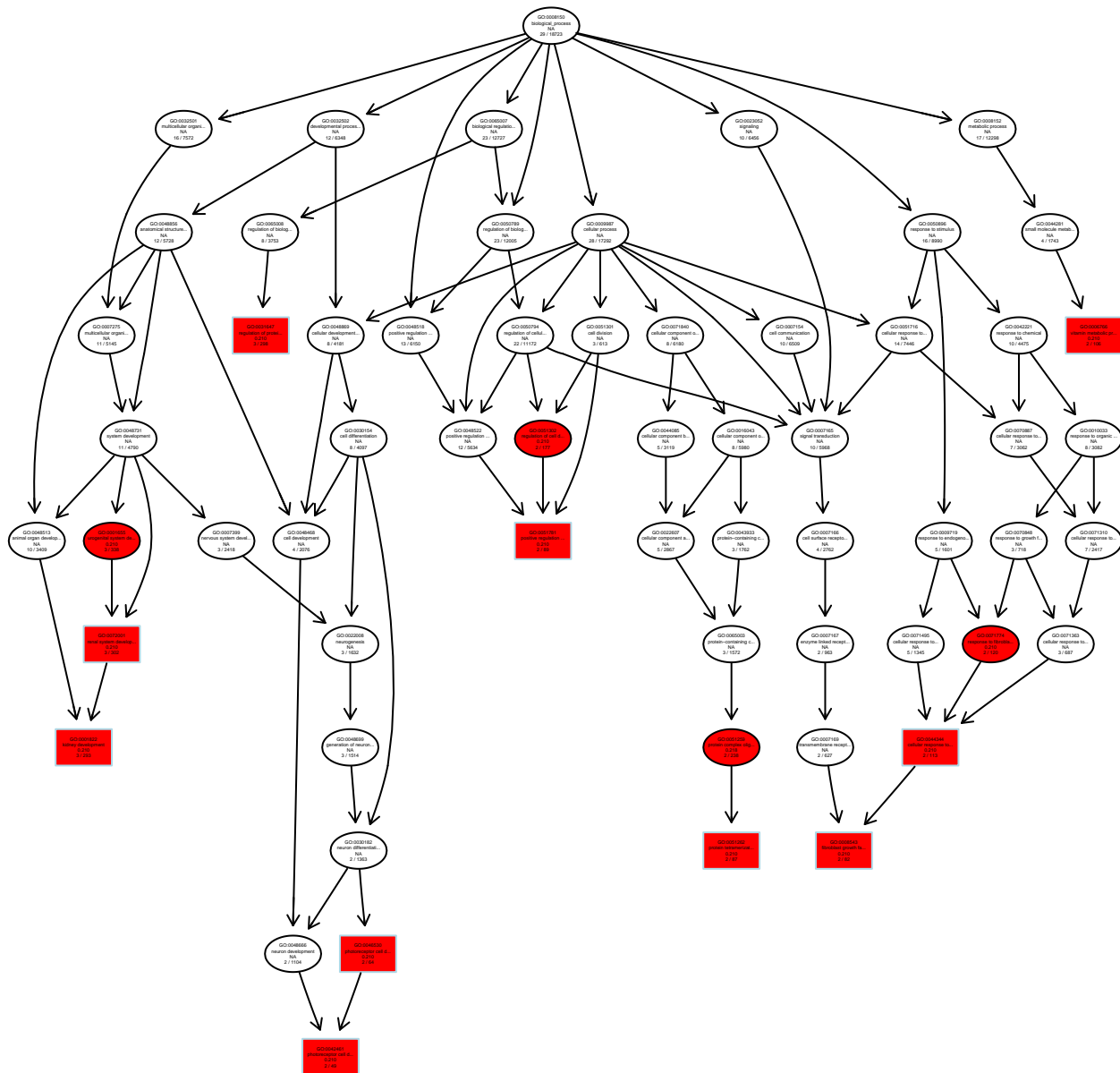

Supplement: Supplementary file 3 — Additional file 3. Figure S3. The GO directed acyclic graphs for the unique biomarker genes of Colorectal cancer group. [file 13148_2024_1646_MOESM3_ESM.pdf]

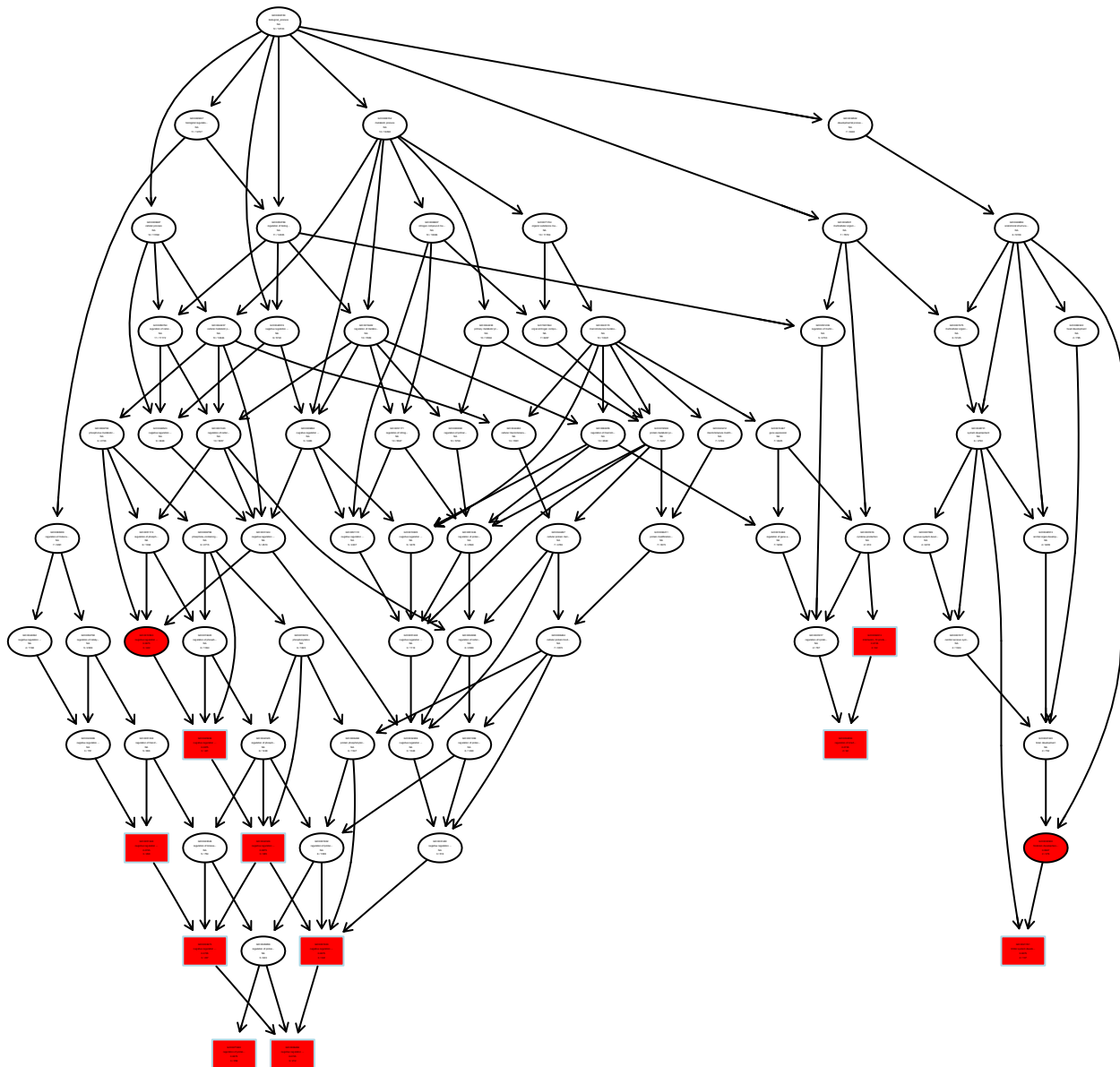

Supplement: Supplementary file 4 — Additional file 4. Figure S4. The GO directed acyclic graphs for the shared biomarker genes among three cancer groups. [file 13148_2024_1646_MOESM4_ESM.pdf]
